# Root biomass and cumulative yield increase with mowing height in *Festuca pratensis* irrespective of *Epichloë* symbiosis

**DOI:** 10.1038/s41598-022-25972-y

**Published:** 2022-12-13

**Authors:** Miika Laihonen, Kalle Rainio, Traci Birge, Kari Saikkonen, Marjo Helander, Benjamin Fuchs

**Affiliations:** 1 Biodiversity Unit, University of Turku, 20014 Turku, Finland; 2 Department of Biology, University of Turku, 20014 Turku, Finland

**Keywords:** Grassland ecology, Agroecology, Plant symbiosis

## Abstract

Increasing agricultural soil carbon sequestration without compromising the productivity of the land is a key challenge in global climate change mitigation. The carbon mitigation potential of grass-based agriculture is particularly high because grasslands represent 70% of the world’s agricultural area. The root systems of grasses transfer large amounts of carbon to below-ground storage, and the carbon allocation to the roots is dependent on the grasses’ photosynthesizing shoot biomass. In a common-garden experiment, *Festuca pratensis* was used as a model species to study how mowing and weed control practices of perennial cool-season fodder grasses affect total yield and root biomass. Additionally, grass-associated *Epichloë* endophytes and soil residual glyphosate were tested for their effect on the total yield and root biomass alone or in interaction with mowing. The results demonstrate that elevating the cutting height increases both cumulative yield and root biomass in *F. pratensis*. Endophyte symbiosis increased the total yield, while glyphosate-based herbicide residues in the soil decreased the root biomass, which indicates a reduction of soil bound carbon sequestration. The findings demonstrate that carbon sequestration and yield quantities on farmed grasslands may significantly be improved by optimizing strategies for the use of plant protection products and adjustment of mowing intensity.

## Introduction

Soils comprise the largest pool of actively cycling carbon in terrestrial ecosystems^1^. Anthropogenic land use change, especially conversion of natural ecosystems to agricultural and urban use, has substantially depleted soil carbon stocks and altered carbon cycles^2^. Increasing soil carbon sequestration in soils with carbon debt is a key component of global climate change mitigation^3^. Globally, approximately 70% of the world’s agricultural area is extensively or intensively managed grasslands^4^. Grasslands are an important potential carbon sink, and approximately 98% of grassland-stored carbon is in the soil^5–7^. This carbon has a slower turnover rate compared to aboveground carbon^8,9^. However, analysis of grasslands globally indicates that greenhouse gas emissions of managed grasslands cancel out the carbon sequestration of sparsely grazed and natural grasslands^10^. Due to the prevalence of perennial grasses in these man-made ecosystems, stimulating soil carbon sequestration via improved grass root growth is a promising tool towards sustainable and climate-wise development goals^6,11–13^.

Developments in mechanization and mowing technologies have led to agricultural intensification and adoption of lower mowing height^14,15^ but the importance of the mowing height to the grass growth has been largely ignored in agricultural practices^16–19^. Although cool-season grasses are adapted to frequent tissue loss^20^, intensive cutting decreases photosynthetic tissues and carbohydrate reserves, which constrain resources needed for regrowth and root growth^15,16,21^. This process affects the carbon sequestration potential of the plants^6,12,22^ because the energy produced through plant photosynthesis is directed firstly to the aboveground meristems, and only then are excess carbohydrates transferred to the carbohydrate reserves and invested into belowground growth^23^.

Agricultural grasslands are managed along a spectrum of low to high intensity. High intensity management entails greater use of inputs and more frequent harvesting. The challenge in high intensity grasslands is to balance agronomic aims of maximized production and weed management with environmental aims of minimizing harm to the environment and increasing soil carbon sequestration.

### Glyphosate-based herbicides

Minimum tillage production is a popular strategy to retain soil organic matter and foster soil carbon sequestration via roots and the rhizodeposition of organic compounds from the living roots to the soil^5,24,25^. However, in high input-based intensive agriculture, field preparation in minimum tillage systems is commonly achieved by the use of glyphosate-based herbicides (GBHs)^26–28^. Since the last glyphosate patents expired in 2000^26^, glyphosate has become the main ingredient of the most widely used contemporary herbicides^27,29^.

Increasing evidence shows that GBHs and their degradation products (mainly aminomethylphosphonic acid, AMPA) can persist in soil longer than previously reported and affect non-target organisms including microbes and crop plants^28,30–35^. The persistence and mobilization of glyphosate in soils are determined by abiotic and biotic environmental conditions^36^. For example, glyphosate is degraded primarily by microbes, and glyphosate and phosphorus are known to compete for the same binding sites in the soils^36–38^. Thus, prolonged persistence of glyphosate residues is commonly detected particularly in northern agroecosystems where microbial soil processes are slower compared to warmer regions^28,39,40^. Although GBHs are not used during the cultivation of perennial grasses, they are commonly applied before establishment or renewal of cultivated fodder fields, and residues in soils can persist following application in GBH-based no-till crop rotation^28,41^.

### Stronger crop with fungal endophytes

Integrated weed and soil management practices such as including forage crops in crop rotation, cover crops, reduction in field cultivation intensity, adjusting harvesting times and use of plant beneficial microbes are promoted as alternatives to chemical weed control^42–45^. In grass production, *Epichloë* fungal endophytes may provide opportunities for integrated pest management and reduce the chemical burden associated with intensive grass production while increasing biomass production^44,46–48^.

*Epichloë* species grow intercellularly and systematically throughout the above-ground plant parts. When growing into host grass inflorescences they are transmitted vertically to the next plant generation via the host plant seeds. Because the fitness of the fungus and the host are tightly linked, *Epichloë*-grass interactions are commonly regarded as mutualistic, and empirical evidence indicates that the fungus can increase stress tolerance, survival and yield in grasses^48–51^. In the USA and New Zealand, *Epichloë* endophytes have been implemented into plant breeding and grass cultivation programs as a means to improve grass production but similar use is not seen in Europe, where the *Epichloë* endophyte frequencies on grasslands and in seed lots are generally low^48,52–55^.

Meadow fescue (*Festuca pratensis*) is a common cool-season (C3) fodder species which exhibits endophytic symbiosis with *Epichloë*. Other characteristics include deep roots and high forage value^56^. Thus, it is a suitable grass species for studying the effects of *Epichloë* symbiosis on the twin aims of production and soil carbon sequestration.

### Study aims

The aim of the study was to determine the effects of GBH, *Epichloë* endophytes and cutting height on plant survival, cumulative yield and plant root biomass in a two-year common-garden experiment using a meadow fescue cultivar. The key assumptions of the study are twofold. Firstly, we assume that glyphosate negatively affects plant growth while endophyte symbiosis positively affects plant growth. Secondly, we assume that the grasses can tolerate and compensate for a moderate level of cutting, but cutting very close to the ground will compromise plant energy reserves, root system development and, subsequently, shoot regrowth. We predict that the mowing height affects cumulative yield (see also^17^) and root biomass, which serves as an approximation for carbon sequestration potential, in grasses during the growing season.

## Results

**Figure 1:**
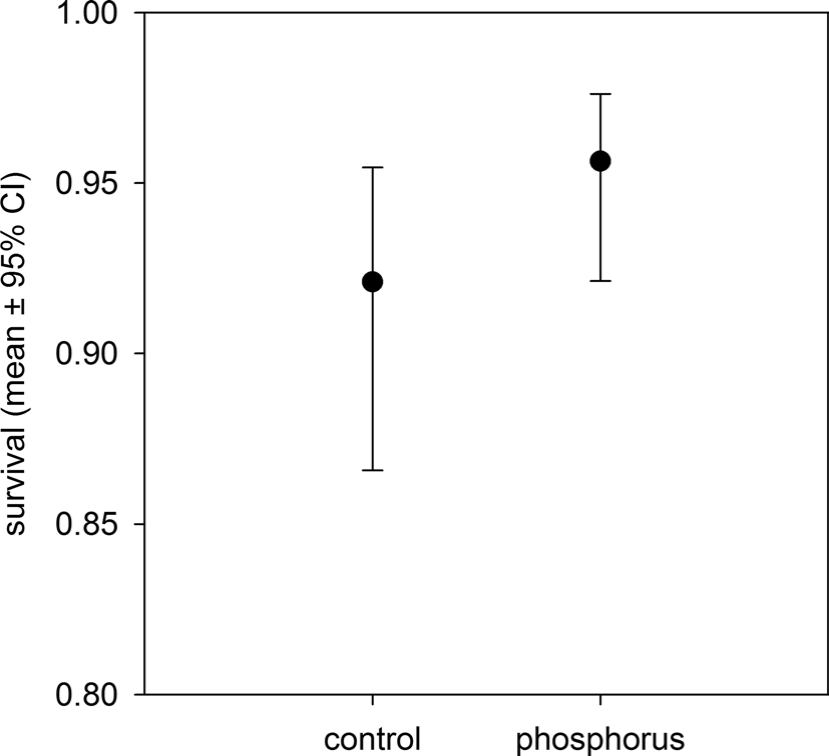
The effect of phosphorus treatment on the survival probability of meadow fescues on the second year of the experiment. The survival in the phosphorus treated plants (96%) was statistically significantly higher (F_1,1150_ = 7.89, p = 0.0051) compared to the control group (92%).

Of all explanatory factors, only phosphorus treatment had a statistically significant effect on plant survival, with extra phosphorus plants exhibiting 96% survival compared to the control group’s 92% (Table 1, Fig. 1).

**Table 1:** Results of a generalized linear mixed model showing effects of phosphorus treatment, endophyte status, glyphosate-based herbicide (GBH) treatment and cutting height (control, 15 cm, 5 cm) on the survival of meadow fescues observed during 2020.

Effect	Num DF	Den DF	F	*p*
Phosphorus treatment	1	1127	8.27	**0.0041**
Endophyte status	1	1127	0.05	0.8256
GBH	1	1127	0.05	0.8246
Cutting height	2	1126	0.19	0.8236

Significant *p*-values are highlighted bold.

**Figure 2:**
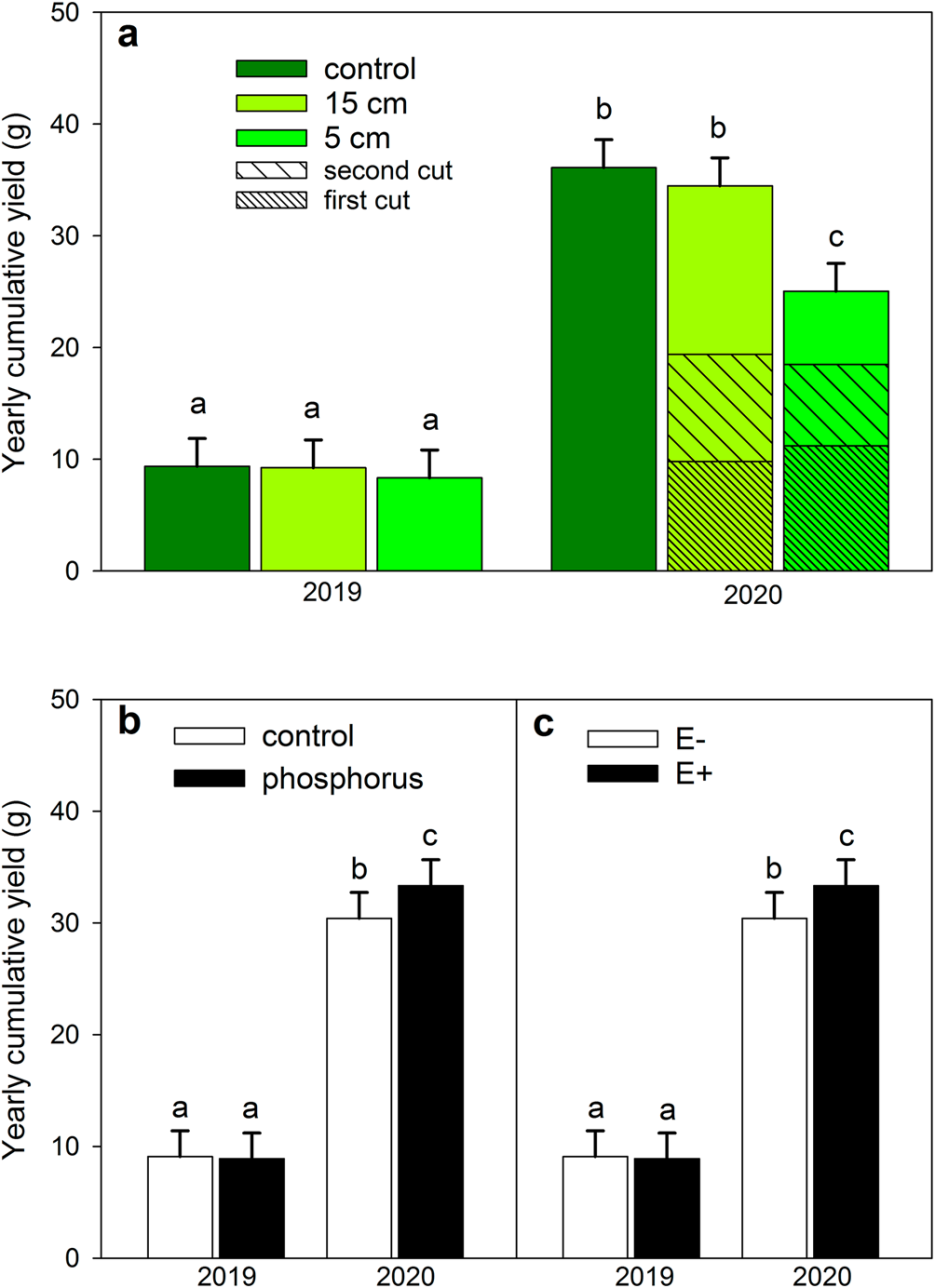
Effect of (**a**) cutting height (control, 15 cm, 5 cm), (**b**) phosphorus treatment and (**c**) endophyte status (E– = endophyte-free, E +  = endophyte-symbiotic) on the yearly cumulative yield (in dry weight) of the meadow fescue in both study years. Data was analyzed with a linear mixed effects model with Tukey–Kramer adjusted pairwise comparisons. Groups sharing a letter do not differ statistically significantly from each other. Error bars represent 95% confidence intervals. N_control_ = 348, N_15 cm_ = 352, N_5 cm_ = 348; N_control_ = 511, N_phosphorus_ = 537; N_E-_ = 523, N_E+_ = 525.

Yearly cumulative yield was affected by the interaction between year and cutting treatment, with a cutting treatment effect only in the second year (Fig. 2a). The aboveground plant biomass of the control (t = − 4.48, *p* < 0.0001) and the 15 cm treatment (t = 6.35, *p* < 0.0001) were both elevated by 44% and 38%, respectively, compared to the plants in the 5 cm treatment during the second growing year (Table 2, Fig. 2a). Furthermore, the effects of phosphorus and endophyte status on plant biomass were only seen in the second year (2020, Table 2, Fig. 2b). Plants in the phosphorus treatment were larger compared to control plants (Fig. 2b, Table 2, second year only: t = − 3.8, *p* = 0.0002). The endophyte symbiotic (E +) plants were larger than the endophyte-free (E–) plants across all treatments (Fig. 2c, Table 2, second year only: t = 2.96, *p* = 0.0032).

**Table 2:** Results of a linear mixed model showing effects of phosphorus treatment, endophyte status, glyphosate-based herbicide (GBH) treatment, cutting height (control, 15 cm, 5 cm), and study year on the yearly cumulative yield of meadow fescues. Only statistically significant interaction terms are presented.

Effect	Num DF	Den DF	F	*p*
Phosphorus treatment	1	539.8	7.11	**0.0079**
Endophyte status	1	539.7	3.90	**0.0489**
GBH	1	22.96	0.11	0.7417
Cutting height	2	539.7	29.32	** < 0.0001**
Year	1	538.8	1082.93	** < 0.0001**
Year * GBH	1	538.8	7.56	**0.0062**
Year * endophyte status	1	538.7	5.01	**0.0256**
Year * cutting height	2	538.7	20.24	** < 0.0001**

Significant *p*-values are highlighted bold.

**Figure 3:**
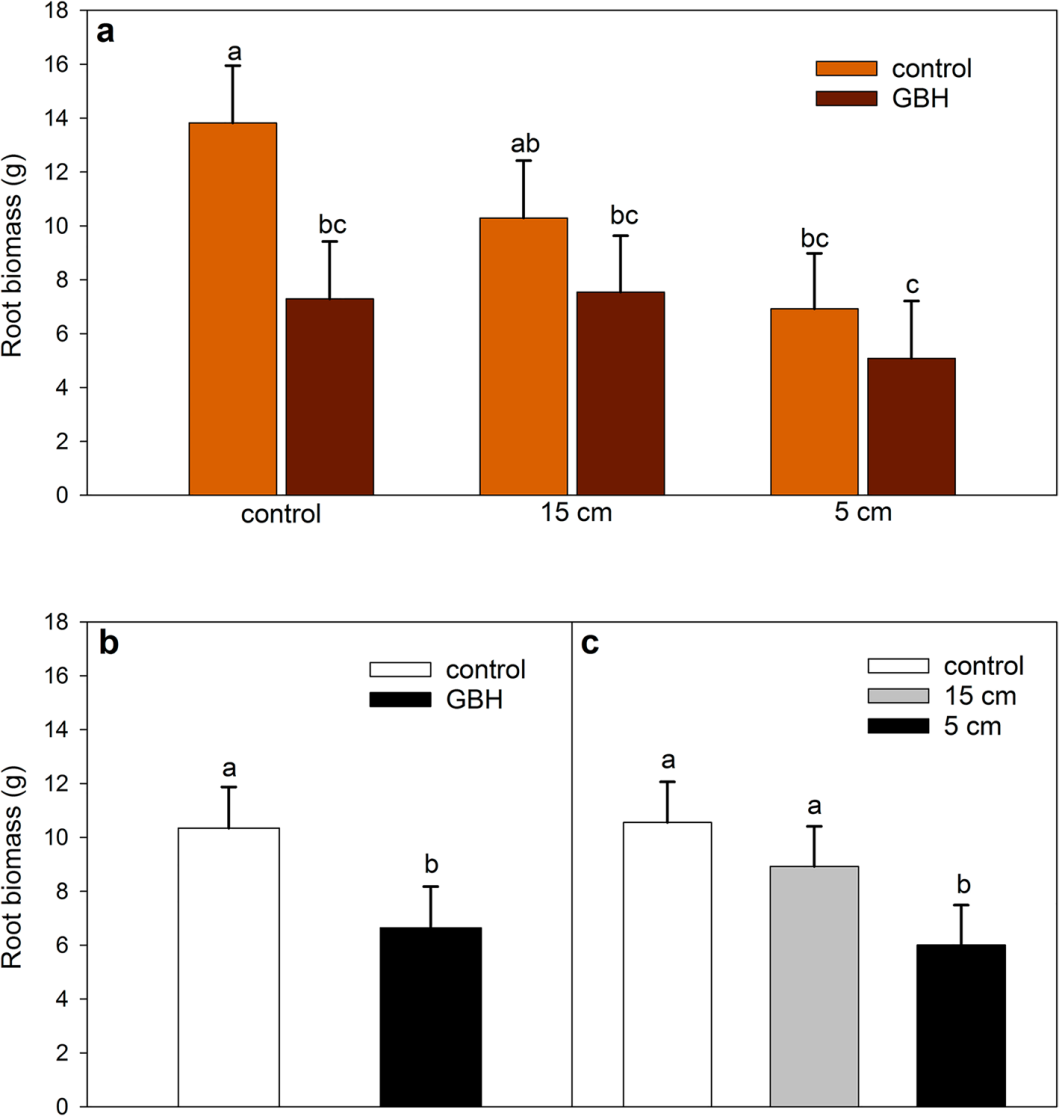
Effect of (**a**) glyphosate-based herbicide (GBH) treatment and cutting height (control, 15 cm, 5 cm) together, (**b**) only GBH treatment and (**c**) only cutting height on the plant root biomass (in dry weight) of the meadow fescue after a two-year experiment. Data was analyzed with a linear mixed effects model with Tukey–Kramer adjusted pairwise comparisons. Groups sharing a letter do not differ significantly from each other. Error bars represent 95% confidence intervals. N_control_ = 72, N_GBH_ = 70; N_control_ = 46, N_15 cm_ = 48, N_5 cm_ = 48.

Root biomass showed a significant interaction between cutting treatment and GBH treatment (F_2,111.8_ = 3.53, *p* = 0.0325, Fig. 3a, Table 3, pairwise comparisons in Table 4). The cutting control plants grown in GBH-treated soil had smaller roots compared to the control soil plants (Fig. 3a). Roots of plants grown in GBH control soil were approximately 6 g heavier than the roots in GBH-treated soil (Fig. 3a). Furthermore, within the GBH controls, 5 cm treatment resulted in smallest roots (Fig. 3a). Cutting height did not have a statistically significant effect on root biomass in plants grown in GBH treated soil (Fig. 3a). The GBH control plants had ~ 3.7 g heavier roots than GBH treated plants (Fig. 3b, Table 3). The 5 cm cutting treatment had a negative effect on root biomass compared to both controls (t = − 4.33, *p* < 0.0001) and the 15 cm cutting treatment (t = 2.72, *p* = 0.0201) (Fig. 3c, Table 3). Endophyte status did not affect the plant root biomass (Table 3).

**Table 3:** Results of a linear mixed model showing effects of endophyte status, glyphosate based herbicide (GBH) treatment and cutting height (5 cm, 15 cm, control) on root biomass of meadow fescue in the end of the two year experiment.

Effect	Num DF	Den DF	F	*p*
Endophyte status	1	113.4	0.27	0.6026
GBH	1	21.01	12.63	**0.0019**
Cutting height	2	111.8	12.28	** < .0001**
GBH * cutting height	2	111.8	3.53	**0.0325**

Statistically non-significant interaction terms are not presented. Significant *p*-values are highlighted bold.

**Table 4:** Statistical analysis of meadow fescue root biomasses with Tukey–Kramer adjusted pairwise comparisons.

Cutting	GBH	Cutting	GBH	DF	t	*p* (adjusted)
**Pairwise comparisons**						
15 cm	GBH	15 cm	Control	2, 124	− 2.03	0.329
15 cm	GBH	5 cm	GBH	2, 124	1.82	0.4577
15 cm	GBH	5 cm	Control	2, 124	0.48	0.9966
15 cm	GBH	Control	GBH	2, 124	0.16	1.0000
15 cm	GBH	Control	Control	2, 124	− 4.62	**0.0001**
15 cm	Control	5 cm	GBH	2, 124	3.81	**0.0029**
15 cm	Control	5 cm	Control	2, 124	2.53	0.1231
15 cm	Control	Control	GBH	2, 124	2.17	0.2589
15 cm	Control	Control	Control	2, 124	− 2.56	0.1154
5 cm	GBH	5 cm	Control	2, 124	− 1.36	0.7524
5 cm	GBH	Control	GBH	2, 124	− 1.64	0.5729
5 cm	GBH	Control	Control	2, 124	− 6.38	** < 0.0001**
5 cm	Control	Control	GBH	2, 124	− 0.32	0.9996
5 cm	Control	Control	Control	2, 124	− 5.15	** < .0001**
Control	GBH	Control	Control	2, 124	− 4.73	** < 0.0001**

Interaction between cutting height (3 groups) and GBH treatment (2 groups) was tested. Significant *p*-values are highlighted bold.

## Discussion

The results demonstrate that an optimized mowing height enhances both biomass production and root size, which is commonly used as approximation for carbon sequestration potential^25^. The grasses cut at the higher cutting regime showed better compensatory regrowth, which overcomes possible temporary yield reduction at first mowing during the harvesting season. The cumulative aboveground biomass was similar in uncut plants and 15 cm cut plants, but the forage quality differed due to development of inflorescences in the uncut plants. Although endophyte symbiosis increased the yield of host grass, it did not affect the root biomass, which suggests that the symbiosis does not foster carbon sequestration in grassland soils as implied in the study by Iqbal et al.^13^. Effects of the GBH were not visible aboveground, but the root development was greatly hindered, which implies negative effects of GBH on carbon sequestration potential.

The results indicate that the mowing practice that increases the carbon sequestration potential in meadow fescue also increases the yield. In their review of the carbon sequestration potential of grasslands, Conant et al.^57^ conclude that management practices introduced with the aim of increasing forage production tend to result in increased soil C stocks. This can result from plant’s increased reserves of excess photosynthates that may be used for root growth^23^. Larger photosynthesizing biomass can potentially transfer more atmospheric C to the roots and, thus, the soil. After an intense mowing event, however, the photosynthetic tissue may lose carbon independence, and soluble carbohydrates in the roots and reserves are then mobilized to compensate for the carbon deficit^58^. This results in a lack of resources for root growth. Our results demonstrate that this decrease in root biomass and the following decrease in carbon sequestration potential to soils^4,6,15,24^ can be avoided by optimizing the mowing height.

Similar to mowing, resource allocation in cool-season grasses may be modulated by the symbiotic *Epichloë* endophytes which compete for the same resources as the plant meristems^51,59,60^. The costs of *Epichloë* endophytes in *F. pratensis* have been detected under low nutrient environments^51^. Here, we detected *Epichloë*-boosted shoot growth irrespective of cutting, phosphorus or GBH treatment. The findings support prior literature suggesting that *Epichloë* endophytes can increase the host growth and photosynthesis, especially in field settings with plant competition^46,53,61–64^. However, in contrast to some earlier findings, no positive effects of *Epichloë* endophytes was found on the root biomass of the host grass^64–66^ and, thus, on carbon sequestration potential in soils^13^. Nevertheless, implementing *Epichloë* endophytes for grassland management optimization can be integrated with plant protection methods to help to reduce the broad use of agrochemicals^48^.

In addition to negative effects of intensive mowing on roots, the results show that GBH greatly decreased root growth, and thus, the carbon sequestration potential to soils. The GBH applied to the soil is in direct contact with the plant roots, can accumulate in root tips and hinder root growth^67,68^. However, GBH treatment did not affect the yield (but see^33^). This may be explained by a fertilizing effect of the phosphorus-containing glyphosate in the soil^27^ or by resources allocated from roots to shoot regrowth^23^. Finding alternative weed management practices would improve the resilience and carbon sequestration potential of grasses.

Due to its fodder value and that it is a deep-rooted species, meadow fescue is a relevant plant species for sustainable grassland production in cool or cold climate conditions. Final root biomass was used as an indirect proxy for soil carbon sequestration potential because measuring carbon sequestration via plant roots during a growing season is difficult due to the constant process of root growth, decomposition and releasing of the root exudates^24,25,69^. Investment in roots and root growth vary in grass species and the outcome may vary in mixed swards in comparison to monocultures. Studies show, for example, that multi species swards have higher C sequestration potential compared to monocultures^10^.

Climate change may also provide the conditions for increased productivity^70^. Climate change is advancing harvesting times and increasing the total yield in northern latitudes^71^. Thus, the importance of grasslands in sequestration of atmospheric carbon is increasing especially in northern latitudes^22,72^. In Finland, for example, an average fodder field yields more than 5.4 tons of dry aboveground biomass per hectare per year^73^, and sequestrates approximately 0.6–0.8 tons of C per hectare in a year^74^. Based on reduced root biomass alone, confident predictions cannot be made on the impact of mowing height on carbon sequestration at agricultural scale. Even though the amount of plant roots largely contributes to carbon sequestration, other factors such as soil type, microbial activity or precipitation need to be considered^6,7^. However, our findings suggest that relatively minor adjustments in mowing height can increase the climate change mitigation potential and overall yield in meadow fescue-based grasslands.

Here, the effects of GBH, *Epichloë* endophytes and cutting height on plant survival, cumulative yield and plant root biomass were studied in a two-year field experiment using a meadow fescue cultivar. An optimized mowing height was found to enhance both biomass production and carbon sequestration potential in plant. Endophyte symbiosis further increased the yield. GBH residues greatly decreased the carbon sequestration potential. This study contributes to the knowledge on response of the growth and associated soil carbon accumulation potential of meadow fescue, which is a relevant species for grass cultivation in cool/cold environments. The findings are in line with the existing principle that excessive cutting can decrease grass yield over multiple harvests. In addition to clarifying the effect of cutting height on yield, the results suggest that both the role of endophytes in improving yield and the impact of glyphosate on soil carbon sequestration are important areas of research for achieving carbon–neutral farming.

## Material and methods

### Study plants and set-up

The widely used meadow fescue (*Festuca pratensis*) cultivar ‘Kasper’ was chosen as a model because the species is commonly used in pastures and as forage in Europe and the cultivar commonly hosts *Epichloë uncinata* endophyte^53^. To study whether the endophyte symbiosis affects the plant tolerance to cutting and, thus, yield and/or root growth, seed-borne offspring collected from 5 endophyte-symbiotic (E +) and 5 endophyte-free (E–) plants was used in the experiment. The endophyte status of the mother plants was verified by microscopically examining 3 seeds per plant. Seeds were chemically softened after which they were prepared for microscoping. In E + plants the fungal hyphae grow between the plant’s embryotic cells (Supplementary Fig. 1) whereas in the E– plants the hyphae are not found (see^75^ for the detailed method).

**Figure 4:**
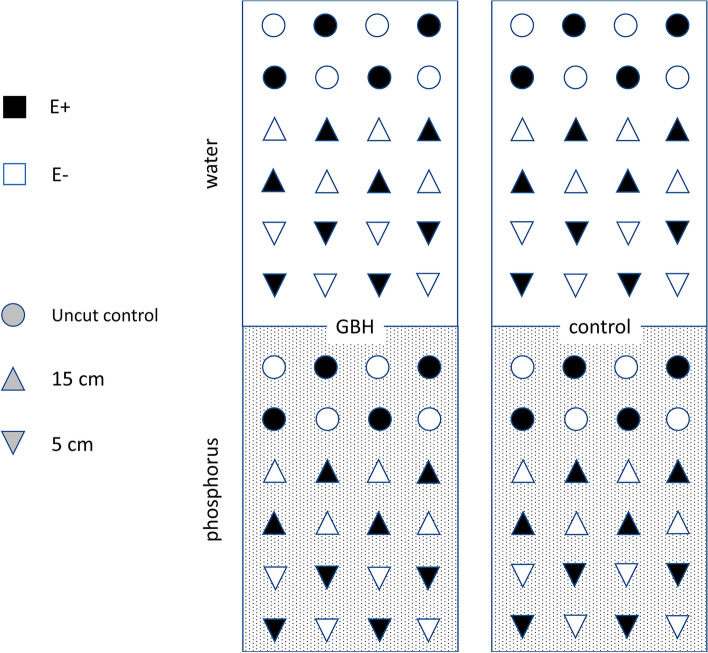
The experimental setup to study the effects of cutting height, phosphorus treatment, glyphosate-based herbicide (GBH) and plant endophyte status (E +  = endophyte-symbiotic, E– = endophyte-free) on plant survival and growth. The figure shows one out of 12 plot pairs with one GBH and one control plot. Three different cutting heights were applied (control, 15 cm, 5 cm) and their orientation alternated between the plots. All plots were divided into two halves: one half was given phosphorus treatment and the other half received only water. 12 E + and 12 E– plants were planted on each half.

The study was carried out on a long-term experimental field simulating no-till farming practices at the Ruissalo Botanical Garden (Turku, Finland, 60° 26′ N, 22° 10′ E). The topsoil of the field consists of approximately 88% clay, 6% sand and 6% peat. Since 2014, the field has been used to study glyphosate-based herbicides (GBH) in 24 plots (23 m × 1.5 m) that were tilled twice a year. Half of the plots are treated twice a year with GBH (RoundUp® Gold; glyphosate concentration 450 g/l; 16.2 ml in three liters of water applied per plot) and the other half (control) sprayed with an equivalent amount of water (see^31^ for further details). The treatment mimics field realistic soil conditions equivalent to application of GBH according to the product instructions. After field treatment, the glyphosate starts to degrade in the soil. To implement the binding site competition between phosphorus and glyphosate in the soil^38^, half of each plot was given extra phosphorus each spring as phosphate (Yara Ferticare™ P-K, 45 kg/ha) diluted in water while the other half of the plot was given an equivalent amount of water (Fig. 4).

A total of 1152 individual *Festuca pratensis* plants were planted on the experimental plots. Planting took place on 4th–5th June 2019 with 10 cm tall seedlings grown from seed in ambient greenhouse conditions. Each half plot had 24 plants (Fig. 4): four endophyte-symbiotic (E +) and four endophyte-free (E–) plants subjected to each cutting treatment. Three cutting treatments were applied to the fescues: (1) eight cut-control plants, where no cutting was applied (2) eight plants were cut to 15 cm, which represents low mowing intensity, and (3) eight plants were cut to 5 cm height, which represents a high mowing intensity. The cutting treatments were conducted once in summer 2019 and twice during summer 2020. The thermic growing season in southernmost Finland lasts on average from the late April to late October. In the end of the growing seasons, October 2019 and September 2020, all plants were cut to the ground level. In each cutting, biomass was pooled from all four plants from the same treatment group per plot. All collected biomasses were pre-dried and oven-dried (65 °C, 15 h) before weighing to the nearest 0.1 g. The yearly cumulative yields were calculated as sums of each cutting during one year.

After the plants had established, in August 2019, soil samples were collected from the plots and sent for GBH residue analysis to Groen Agro [https://agrocontrol.nl/en/home-en/]). The GBH–treated plots contained an average of 65 μg/kg of glyphosate and 1850 μg/kg of AMPA residues. Any potential residues in the control plots were below detectable limits (glyphosate: ≤ 10 μg/kg, AMPA: ≤ 50 μg/kg).

In September 2020, root samples were taken from one randomly chosen plant per treatment combination (endophyte x clipping) per plot. Plants on phosphorus-treated plots were excluded for a total of 144 root samples. Whole plants were dug up, roots were washed and cut from the base, pre-dried and finally oven-dried (65 °C, 15 h) before being weighed to the nearest 0.1 g.

In May 2020, GBH was carefully applied as a solution around the growing fescues while avoiding direct exposure to the leaves. The dose of the GBH was calculated to mimic the spraying during May 2019 as closely as possible. The control plants were similarly treated with water. The phosphorus was applied following the described method for 2019. Plant survival was monitored once per year.

### Statistical analyses

The survival of experimental plants (yes/no) was analyzed with a generalized linear mixed model with the explanatory variables cutting treatment, endophyte status and phosphorus and glyphosate treatment. Non-significant interactions were dropped from the final model. Since dead plants were not replaced, only the survival recorded at the end of the study in 2020 was considered. Plot was included as a random effect in the model.

The cumulative aboveground biomass (yield) per season (corrected with the number of live plants) was analyzed with a linear mixed effects model with the explanatory variables year, cutting treatment, endophyte status, phosphorus and GBH treatment and all interaction terms between the explanatory variables. Plot was included as a random effect in the model. The plant root biomass was analyzed with a linear mixed effects model with the explanatory variables cutting treatment, endophyte status, GBH treatment and all interaction terms between the explanatory variables. Plot was included as a random effect in the model. A Tukey–Kramer adjusted post-hoc test was conducted to test the pairwise differences in aboveground biomasses between the cutting treatment groups and the pairwise differences in root biomasses between the groups of cutting treatment—GBH treatment interaction term. Non-significant interactions were dropped from the final models. All statistical analyses were performed with proc Glimmix of the SAS 9.4 statistical software package.

### Permissions and/or licenses for collection of plant material

All methods were performed in accordance with the relevant guidelines/regulations/legislation. The *Festuca pratensis* cultivar used is commercially available in Finland. All sampled plants were grown during the experiment and no permissions or licenses were required to collect or use the plant material on which the data is based.

## Supplementary Information

Supplementary Information.

## Data Availability

The datasets generated during and/or analyzed during the current study are available from the corresponding author upon request.
