# Supplementary material for: Root biomass and cumulative yield increase with mowing height in Festuca pratensis irrespective of Epichloë symbiosis
Source: Sci Rep. 2022 Dec 13;12:21556. doi: 10.1038/s41598-022-25972-y (PMC9748035; doi:10.1038/s41598-022-25972-y)
Supplement: Supplementary file 1 — Supplementary Information. [file 41598_2022_25972_MOESM1_ESM.pdf]

Root biomass and cumulative yield increase with mowing height in *Festuca pratensis* irrespective of *Epichloë* symbiosis

Laihonen, Miika[1], Rainio, Kalle[1], Birge, Traci[1], Saikkonen, Kari[1], Helander, Marjo[2], and Fuchs, Benjamin[1*]

[1]Biodiversity Unit, University of Turku, 20014, Turku, Finland; [2]Department of Biology, University of Turku, 20014, Turku, Finland

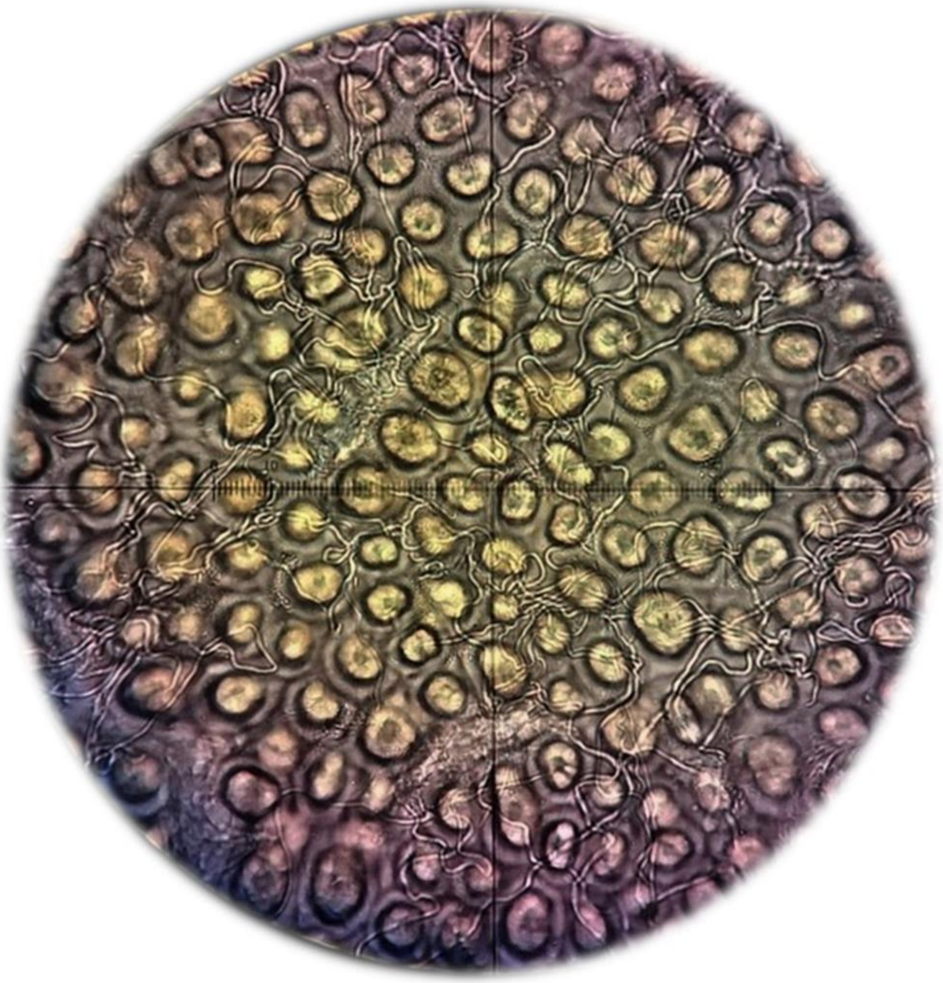

Supplementary figure 1. Hyphae of endophyte growing between the embryotic cells in tall fescue seed as seen through the microscope. By microscopically examining its seeds, it is possible to identify if the plant individual is Epichloë-symbiotic or not. Hyphae like this is not present in the seeds without the Epichloë.
